# Supplementary material for: MiRNA let-7 from TPO(+) Extracellular Vesicles is a Potential Marker for a Differential Diagnosis of Follicular Thyroid Nodules
Source: Cells. 2020 Aug 18;9(8):1917. doi: 10.3390/cells9081917 (PMC7465637; doi:10.3390/cells9081917)
Supplement: Supplementary file 1 [file cells-09-01917-s001.pdf]

Zabegina L. et all., MiRNA let-7 from TPO(+) extracellular vesicles is a potential marker for a differential diagnosis of follicular thyroid nodules

Table S1. Oligonucleotides used for the RT-qPCR analysis of miRNA.

**miRNA**	**hsa-let-7a-5p**
Sequence	ugagguaguagguuguauaguu
Stem-looped RT	GTCGTGTCTGAGGCTCACTGACACAATTCGCACCCTCGACACGACAACTATAC
FW primer	CAGCACTGAGGTAGTAGGTT
RV primer	CTGAGGCTCACTGACACAA
Probe	(R6G)-C+C+C+T+CGA+CACGACAACTATAC-(BHQ1)
**miRNA**	**hsa-let-7b-5p**
Sequence	ugagguaguagguugugugguu
Stem-looped RT	GTCGTGTCTGAGGCTCACTGACACAATTCGCACCCTCGACACGACAACCACAC
FW primer	ACAGCACTGAGGTAG+TAGGT
RV primer	CTGAGGCTCACTGACACAA
Probe	(R6G)-C+T+CGA+CA+CGACAACCACAC-(BHQ1)
**miRNA**	**hsa-let-7d-5p**
Sequence	agagguaguagguugcauaguu
Stem-looped RT	GTCGTGTCTGAGGCTCACTGACACAATTCGCACCCTCGACACGACAA+C+TATGC
FW primer	CAGCACAGAGGTAGTAGGTT
RV primer	CTGAGGCTCACTGACACAA
Probe	(R6G)-T+TCGCACCC(T-BHQ1)CGACACGACAACTATGC-p
**miRNA**	**hsa-let-7e-5p**
Sequence	ugagguaggagguuguauaguu
Stem-looped RT	GTCGTGTCTGAGGCTCACTGACACAATTCGCACCCTCGACACGACAACTATAC
FW primer	CAGCACTGAGGTAGGAGGTT
RV primer	CTGAGGCTCACTGACACAA
Probe	(R6G)-CG+CA+C+CC(T-BHQ1)CGACACGACAACTATAC-p
**miRNA**	**hsa-let-7f-5p**
Sequence	ugagguaguagauuguauaguu
Stem-looped RT	GTCGTGTCTGAGGCTCACTGACACAATTCGCACCCTCGACACGACAACTATAC
FW primer	CAGCACTGAGGTAGTAGA+TT
RV primer	CTGAGGCTCACTGACACAA
Probe	(R6G)-C+CC+T+CGA+CA+CGACAACTATAC-(BHQ1)
**miRNA**	**hsa-let-7g-5p**
Sequence	ugagguaguaguuuguacaguu
Stem-looped RT	GTCGTGTCTGAGGCTCACTGACACAATTCGCACCCTCGACACGACAACTGTAC
FW primer	CAGCACTGAGGTAGTAGT+TTG
RV primer	CTGAGGCTCACTGACACAA
Probe	(R6G)-T+TCGCACCC(T-BHQ1)CGACACGACAACTGTAC-p
